# Effect of ginger-based zingibain enzyme on growth and intestinal health in Japanese quails

**DOI:** 10.5194/aab-67-571-2024

**Published:** 2024-12-12

**Authors:** Asad Sultan, Tariq Aziz, Ziaul Islam, Muhammad Shahkar Uzair, Ibrahim Abdullah Alhindary, Rifat Ullah Khan, Ruchi Tiwari

**Affiliations:** 1 Department of Poultry Science, Faculty of Animal Husbandry and Veterinary Sciences, The University of Agriculture, Peshawar, Pakistan; 2 Department of Basic and Applied Zoology, Shaheed Benazir Bhutto University, Sheringal, Upper Dir, Pakistan; 3 Department of Animal Production, College of Food and Agriculture Science, King Saud University, P.O. Box 2455, Riyadh 11451, Saudi Arabia; 4 College of Veterinary Sciences, Faculty of Animal Husbandry and Veterinary Sciences, The University of Agriculture, Peshawar, Pakistan; 5 Department of Veterinary Microbiology, College of Veterinary Science & Animal Husbandry DUVASU, Mathura-281001, UP, India

## Abstract

This study investigates the effects of a ginger-derived phyto-protease enzyme on the growth performance, feed efficiency, carcass quality, intestinal histomorphology, and nutrient digestibility of Japanese quails fed an animal protein concentrate (APC)-based diet. A total of 400 day-old Japanese quail chicks were randomly assigned to four dietary treatments, including a control group (standard diet without phyto-protease) and three experimental groups with varying levels of ginger phyto-protease (0 %, 0.01 %, and 0.02 %) in their APC-based diets. Over a 42 d experimental period, significant ( P<0.01 ) improvements were observed in feed intake, body weight gain, feed conversion ratio (FCR), dressing percentage, and intestinal villus height and width in the groups supplemented with ginger phyto-protease, particularly at the 0.02 % inclusion level. Nutrient digestibility, especially for dry matter, crude protein, and fiber, was also significantly ( P<0.01 ) enhanced in the supplemented groups. These findings suggest that the inclusion of ginger-derived phyto-protease at an optimal rate of 0.02 % in APC-based diets can significantly improve the overall performance and health of Japanese quails.

## 1 Introduction

Optimizing growth, feed efficiency, and gut health in Japanese quails requires balancing diets, utilizing supplements, and maintaining strict management practices (Ahmad et al., 2023a; Anwar et al., 2023a; Gul and Alsayeqh, 2022a, b; Hassan et al., 2023). These strategies lead to improved weight gain, feed efficiency, and resilience (Imran and Alsayeqh, 2022; Hussain et al., 2023; Abbas and Alkheraije, 2023; Al-Hoshani et al., 2023). In the search for natural alternatives, bioactive compounds from plants, like zingibain from ginger, show potential for enhancing nutrient digestion and health (Shah et al., 2023; Kausar et al., 2024).

Zingibain, a proteolytic enzyme in ginger, has gained attention for its potential to improve nutrient absorption and gut health in poultry diets. Its therapeutic properties, such as anti-inflammatory and wound-healing effects, have been explored in biomedical research (Choi et al., 2016; Khan et al., 2012; Raza et al., 2016; Safiullah et al., 2019). Zingibain's role in enhancing the bioavailability of compounds in herbal medicines further underscores its diverse applications (Huang et al., 2018). It improves nutrient absorption by breaking down proteins into peptides and amino acids, supporting efficient feed conversion and promoting growth and health in poultry (Rahman et al., 2018; Gupta et al., 2020). Moreover, zingibain's proteolytic activity enhances protein digestibility and nutrient absorption (Tan et al., 2017; Jabbar et al., 2021). Research in other sectors, like meat processing and pharmaceuticals, has also demonstrated zingibain's utility. Nair et al. (2019) showed its effectiveness in improving meat tenderness and flavor in beef. Additionally, Tan et al. (2017) explored zingibain in pharmaceuticals for enzyme replacement therapy due to its therapeutic benefits.

Despite its potential, zingibain has yet to see widespread use in poultry feed formulations, presenting an opportunity for further research and practical application. This study addresses this by evaluating ginger-derived liquid phyto-protease, specifically zingibain, in Japanese quails (*Coturnix japonica*). The objective of this study was to evaluate the effects of a ginger-derived phyto-protease enzyme on the growth performance, feed efficiency, carcass quality, intestinal histomorphology, and nutrient digestibility of Japanese quails fed an animal protein concentrate (APC)-based diet.

**Table 1:** Basal feed diet composition of the quails.

Ingredients	CON	N-CON	CON	N-CON
	Starter phase (day 1–21)		Finisher phase (day 22–42)	
Corn	40.09	40.09	59.75	59.75
Rice polishing	0.00	0.00	15.00	15.00
Soybean meal 44 % product	37.62	33.62	10.53	6.53
PBM	0.00	4.00	0.00	4.00
Rapeseed meal	6.00	6.00	6.00	6.00
Corn gluten 60 %	5.00	5.00	0.00	0.00
PBM/APC high fat	4.00	4.00	5.00	5.00
Vegetable oil	3.60	3.60	0.23	0.23
Limestone/chips	1.80	1.80	1.52	1.52
Monocalcium phosphate	0.77	0.77	0.00	0.00
Dicalcium phosphate	0.00	0.00	0.29	0.29
DL-methionine	0.33	0.33	0.29	0.29
Salt	0.24	0.24	0.27	0.27
L-threonine	0.23	0.23	0.34	0.34
Lysine sulfate	0.09	0.09	0.41	0.41
L-tryptophan	0.00	0.00	0.07	0.07
Vitamin premix	0.05	0.05	0.05	0.05
Choline chloride	0.05	0.05	0.05	0.05
Salinomycin sodium	0.00	0.00	0.05	0.05
Mineral premix	0.00	0.00	0.05	0.05
Enramycin 4 %	0.02	0.0200	0.02	0.02
Diclazuril 1 %	0.01	0.0100	0.00	0.00
Antioxidant (LOX)	0.01	0.0100	0.01	0.01
Axtra PHY GOLD 1	0.01	0.0100	0.01	0.01
Chemical analysis				
Crude protein (%)	22.0	22.0	21.0	21.0
Metabolizable energy (Kcal kg^−1^)	2800	2800	2900	2900
Crude fiber (%)	3.0	3.0	3.5	3.5
Lysine (%)	1.3	1.3	1.3	1.3
Calcium (%)	0.95	0.95	0.84	0.86
Methionine + cysteine (%)	0.82	0.82	0.71	0.73
Threonine (%)	0.87	0.88	0.61	0.62
Available phosphorus (%)	0.42	0.46	0.46	0.44
Sodium (%)	0.21	0.21	0.17	0.14
Chloride (%)	0.21	0.22	0.17	0.15

PBM: poultry by-product meal. APC: animal protein concentrate.

**Table 2:** Composition of animal protein concentrate (APC).

Nutrients' composition	%
Crude protein	62.22–64.54
Ash content	14.78–16.23
Moisture (%)	5.12–7.43
Fats	15.32–17.51
Ether extract (%)	12.45–13.04
Calcium (%)	3.89–4.49
Phosphorus (%)	2.29–2.59
Crude fiber (%)	1.22–1.42
Gross energy (Kcal kg^−1^)	2800–3000

## 2 Materials and methods

### 2.1 Experimental birds and housing

A total of 400 day-old Japanese quail chicks were purchased from a local market and housed under controlled conditions. The quails were randomly divided into four groups (with five replicates). During the 42 d trial period, the birds had free access to clean water and feed (Tables 1 and 2) following National Research Council (1994) guidelines. They were kept in floor pens with wood shavings as bedding and were given 24 h light. Strict biosecurity measures were in place to prevent disease outbreaks, such as disinfecting facilities, controlling farm access, and maintaining hygiene standards, ensuring flock health and improved productivity.

### 2.2 Source of phyto-protease

The ginger-derived liquid phyto-protease was obtained from a newly funded protein analysis laboratory by the Pakistan Science Foundation (PSF). The extraction process, based on Nafi et al. (2014), involved thoroughly washing ginger rhizomes, air-drying them for 24 h, and finely chopping them. The chopped rhizomes were blended with potassium phosphate buffer, filtered, and centrifuged. Protease activity was determined using azocasein as the substrate and measured spectrophotometrically at 410 nm. The specific activity of the protease was expressed in enzyme units per milligram of protein, following precise enzymatic activity protocols.

### 2.3 Feed preparation

The experimental diets were formulated and produced at a local feed mill. The control group (CON) was given a standard commercial quail diet without protease, while three treatment groups received 4 % animal protein concentrate (APC), partially replacing soybean meal. The groups were divided as follows:

- CON (T1) – standard diet, no phyto-protease;
- T2 – 4 % APC, no phyto-protease;
- T3 – 4 % APC with 0.01 % ginger phyto-protease;
- T4 – 4 % APC with 0.02 % ginger phyto-protease.

Diets were prepared using Brill Formulation^®^ software and transported in sealed bags to ensure freshness and avoid contamination.

### 2.4 Growth performance

Feed intake was calculated by measuring the amount of feed provided and recording the leftover feed daily to determine the average feed intake per bird. Weekly body weights were measured using digital scales to determine the average weight gain per bird. The feed conversion ratio (FCR) was calculated weekly by dividing the total feed intake by the total weight gain of the birds. At the end of the experiment, five birds per replicate were randomly selected for slaughter. Their live weights were recorded before they were slaughtered and dressed. The weights of the carcass, liver, heart, gizzard, pancreas, and abdominal fat were recorded. The dressing percentage was calculated as follows: dressing percentage = (carcass weight/Live bird weight) ×100 .

### 2.5 Intestinal histomorphology

Three birds per replicate were euthanized, and a 3 cm section of ileal tissue was collected. The tissues were gently rinsed with a saline solution; fixed in 10 % buffered formalin for 4 h; and processed for histopathological examination to evaluate villus height, crypt depth, and overall intestinal structure.

### 2.6 Determination of apparent ileal nutrient digestibility 

On day 38, five birds per replicate were transferred to metabolic cages for a 4 d fecal collection period. Feed intake and fecal output were recorded daily to assess nutrient digestibility. The carcasses were dissected, and the ileum was collected and kept chilled for chemical analysis. After freeze-drying, the dry matter, crude protein, ether extract, crude fiber, and apparent metabolizable energy levels of the ileal digesta samples were measured using the methodology described by Hafeez et al. (2020).

$$\begin{array}{rl} \text{apparent} & \text{ileal digestibility}\;(\%)=100\\ & -\frac{\text{conc. of marker in feed }}{\text{conc. of marker in digesta }}\\ & \times \frac{\text{conc. of nutrient in digesta }}{\text{conc. of nutrient in feed}}\times 100 \end{array}$$

### 2.7 Economics

The following formula was used to compare the economic status of birds among various treatments.

$$\begin{array}{c} \begin{array}{rl} \text{feed cost} & \;\text{(in grams) per kilogram live weight}=\\ & \text{cost of feed per bird:}\text{live weight of each bird} \end{array}\\ \begin{array}{rl} \text{gross profit} & =\text{market price (per kg)}\times \text{live weight}\\ & \text{in kilograms (in grams)} \end{array}\\ \begin{array}{rl} \text{income over feeding costs} & =\text{gross return}\\ & -\text{feed cost per chick}. \end{array} \end{array}$$

## 3 Statistical analysis

Data on gut health, growth performance, and nutrient utilization of quails were analyzed using a complete randomized design (CRD) for analysis of variance (ANOVA) with repeated measures for multiple observations over time. Differences among means were compared at a 5 % level of significance using Tukey's test for multiple comparisons. Further statistical analyses were performed using SPSS 21.0 software. Additionally, orthogonal contrast was also used to find the effect of different levels of phyto-protease enzyme concentration on studied parameters.

**Table 3:** Impact of ginger-derived phyto-protease enzyme on feed intake (g per bird) for birds fed an APC-based diet.

Group	T1	T2	T3	T4	P value	Orthogonal contrast	
Week 1	42.00 ± 0.61	41.00 ± 0.57	42.00 ± 0.60	42.00 ± 0.57	0.55	Linear	0.63
						Quadric	0.54
Week 2	71.00^b^ ± 0.60	68.66^c^ ± 0.63	73.00^ab^ ± 0.57	75.00^a^ ± 0.57	0.001	Linear	0.01
						Quadric	0.01
Week 3	81.00^b^ ± 0.57	78.66^c^ ± 0.60	83.00^b^ ± 0.57	86.00^a^ ± 0.57	0.001	Linear	0.01
						Quadric	0.01
Starter	194.0^c^, ± 0.58	188.3^d^ ± 0.63	198.0^b^ ± 0.55	203.0^a^ ± 0.57	0.001	Linear	0.01
						Quadric	0.01
Week 4	112.0^c^ ± 0.57	109.0^d^ ± 0.58	115.0^b^ ± 0.57	118.0^a^ ± 0.59	0.001	Linear	0.01
						Quadric	0.01
Week 5	121.0^c^ ± 0.57	117.0^d^ ± 0.56	124.0^b^ ± 0.57	127.0^a^ ± 0.55	0.001	Linear	0.01
						Quadric	0.01
Week 6	129.0^c^ ± 0.57	123.0^d^ ± 0.57	134.0^b^ ± 0.57	136.0^a^ ± 0.57	0.001	Linear	0.01
						Quadric	0.01
Finisher	362.0^c^ ± 0.57	349.0^d^ ± 0.61	373.0^b^ ± 0.60	381.0^a^ ± 0.57	0.001	Linear	0.01
						Quadric	0.01
Total	556.0^c^ ± 0.64	537.0^d^ ± 0.63	571.0^b^ ± 0.62	584.0^a^ ± 0.60	0.001	Linear	0.01
						Quadric	0.01

Mean values bearing different superscripts in a row differ significantly ( P<0.05 ). CON: standard diet with no phyto-protease. T2: 4 % APC diet with no phyto-protease. T3: 4 % APC diet supplemented with 0.01 % ginger phyto-protease. T4: 4 % APC diet supplemented with 0.02 % ginger phyto-protease.

## 4 Results

Table 3 presents the impact of ginger-derived phyto-protease enzyme on feed intake (g per bird) for birds fed an APC-based diet. During the first week (W1), there was no significant difference in feed intake among the groups, with values consistently around 41–42 g per bird ( P=0.5523 ). However, in the second week (W2), significant differences emerged ( P=0.0011 ). Group T4 had the highest feed intake at 75.00 g per bird, followed by T3 with 73.00 g per bird, T1 with 71.00 g per bird, and T2 with the lowest intake at 68.66 g per bird.

By the third week (W3), these differences became more pronounced ( P=0.0003 ), with Group T4 again leading in feed intake at 86.00 g per bird, followed by T3 at 83.00 g per bird, T1 at 81.00 g per bird, and T2 at 78.66 g per bird. During the starter phase, a significant variation in feed intake was observed ( P=0.001 ), where Group T4 had the highest intake at 203.0 g per bird, followed by T3 at 198.0 g per bird, T1 at 194.0 g per bird, and T2 at 188.3 g per bird.

In the fourth week (W4), feed intake continued to show significant differences among groups ( P=0.001 ). Group T4 maintained the highest intake at 118.0 g per bird, followed by T3 at 115.0 g per bird, T1 at 112.0 g per bird, and T2 at 109.0 g per bird. This trend persisted into the fifth week (W5), where Group T4 again had the highest intake at 127.0 g per bird, T3 at 124.0 g per bird, T1 at 121.0 g per bird, and T2 at 117.0 g per bird, with the differences being statistically significant ( P=0.001 ).

By the sixth week (W6), the significant variation in feed intake among groups remained ( P=0.001 ). Group T4 led with 136.0 g per bird, followed by T3 with 134.0 g per bird, T1 with 129.0 g per bird, and T2 with 123.0 g per bird. During the finisher phase, feed intake showed significant differences ( P=0.001 ), with Group T4 having the highest intake at 381.0 g per bird, followed by T3 at 373.0 g per bird, T1 at 362.0 g per bird, and T2 at 349.0 g per bird.

Overall, total feed intake was significantly higher in the groups supplemented with the ginger-derived phyto-protease enzyme ( P=0.001 ). Group T4 had the highest total intake at 584.0 g per bird, followed by T3 at 571.0 g per bird, T1 at 556.0 g per bird, and T2 at 537.0 g per bird. The data clearly indicated that the inclusion of the ginger-derived phyto-protease enzyme significantly enhanced feed consumption, particularly in the groups with higher enzyme levels (T3 and T4), demonstrating the enzyme's potential efficacy in improving feed intake in birds. Except for week 1, a linear and quadratic response was observed for weekly feed intake, as well as for starter, finisher, and total feed intake in quails.

**Table 4:** Impact of ginger-derived phyto-protease enzyme on body weight gain (g per bird) for birds fed an APC-based diet.

Group	T1	T2	T3	T4	P value	Orthogonal contrast	
Week 1	13.00 ± 0.57	11.66 ± 0.60	13.66 ± 0.58	13.00 ± 0.57	0.085	Linear	0.97
						Quadric	0.11
Week 2	21.00^bc^ ± 0.59	20.00^c^ ± 0.57	22.33^ab^ ± 0.58	23.33^a^ ± 0.59	0.004	Linear	0.01
						Quadric	0.01
Week 3	23.00^c^ ± 0.58	21.66^c^ ± 0.61	25.00^b^ ± 0.57	27.00^a^ ± 0.57	0.001	Linear	0.01
						Quadric	0.01
Starter	57.00^a^ ± 0.60	53.33^c^ ± 0.58	61.33^a^ ± 0.61	63.33^a^ ± 0.57	0.001	Linear	0.01
						Quadric	0.01
Week 4	34.33^b^ ± 0.57	32.00^c^ ± 0.59	36.66^a^ ± 0.61	38.00^a^ ± 0.57	0.001	Linear	0.01
						Quadric	0.01
Week 5	34.66^b^ ± 0.59	32.33^c^ ± 0.62	36.00^b^ ± 0.61	38.00^a^ ± 0.57	0.001	Linear	0.01
						Quadric	0.01
Week 6	38.00^b^ ± 0.56	35.00^c^ ± 0.57	40.33^a^ ± 0.58	42.00^a^ ± 0.56	0.001	Linear	0.01
						Quadric	0.01
Finisher	107.0^c^ ± 0.58	99.33^d^ ± 0.60	113.0^b^ ± 0.57	118.0^a^ ± 0.59	0.001	Linear	0.01
						Quadric	0.01
Total	164.00^c^ ± 0.57	152.67^d^ ± 0.58	174.00^b^ ± 0.57	181.33^a^ ± 0.60	0.001	Linear	0.01
						Quadric	0.01

Mean values bearing different superscripts in a row differ significantly ( P<0.05 ). CON: standard diet with no phyto-protease. T2: 4 % APC diet with no phyto-protease. T3: 4 % APC diet supplemented with 0.01 % ginger phyto-protease. T4: 4 % APC diet supplemented with 0.02 % ginger phyto-protease.

The impact of ginger-derived phyto-protease enzyme on body weight gain (g per bird) for birds fed an APC-based diet is given in Table 4. In the first week (W1), there was no significant difference in body weight gain among the groups, with values ranging from 11.66 to 13.66 g per bird ( P=0.0855 ). By the second week (W2), significant differences emerged ( P=0.0049 ), with Group T4 achieving the highest body weight gain at 23.33 g per bird, followed by T3 at 22.33 g per bird, T1 at 21.00 g per bird, and T2 at 20.00 g per bird.

In the third week (W3), the differences became more pronounced ( P=0.0005 ), with Group T4 again leading in body weight gain at 27.00 g per bird, followed by T3 at 25.00 g per bird, T1 at 23.00 g per bird, and T2 at 21.66 g per bird. During the starter phase, there was a significant variation in body weight gain ( P=0.0003 ), where Group T4 had the highest gain at 63.33 g per bird, followed closely by T3 at 61.33 g per bird, T1 at 57.00 g per bird, and T2 at 53.33 g per bird.

In the fourth week (W4), the significant variation in body weight gain continued ( P=0.0001 ). Group T4 led with 38.00 g per bird, followed by T3 with 36.66 g per bird, T1 with 34.33 g per bird, and T2 with 32.00 g per bird. This trend persisted into the fifth week (W5), where Group T4 again had the highest gain at 38.00 g per bird, T3 at 36.00 g per bird, T1 at 34.66 g per bird, and T2 at 32.33 g per bird, with the differences being statistically significant ( P=0.0002 ).

During the fifth week, the feed intake of Japanese quails fed an APC-based diet was significantly affected by the ginger-derived phyto-protease enzyme (Table 3). Group T4 had the highest feed intake at 127.0 g per bird ( ± 0.55), followed by group T3 at 124.0 g per bird ( ± 0.57) and group T1 at 121.0 g per bird ( ± 0.57). The lowest feed intake was observed in group T2 at 117.0 g per bird ( ± 0.56). The differences between the groups were statistically significant ( p=0.001 ), with both linear and quadratic contrasts showing significance ( p=0.01 ).

By the sixth week (W6), the significant differences in body weight gain among groups remained ( P=0.0001 ). Group T4 had the highest gain at 42.00 g per bird, followed by T3 at 40.33 g per bird, T1 at 38.00 g per bird, and T2 at 35.00 g per bird. During the finisher phase, body weight gain showed significant differences ( P=0.0003 ), with Group T4 having the highest gain at 118.0 g per bird, followed by T3 at 113.0 g per bird, T1 at 107.0 g per bird, and T2 at 99.33 g per bird.

Overall, total body weight gain was significantly higher in the groups supplemented with the ginger-derived phyto-protease enzyme ( P=0.0001 ). Group T4 had the highest total gain at 181.33 g per bird, followed by T3 at 174.00 g per bird, T1 at 164.00 g per bird, and T2 at 152.67 g per bird. The data clearly indicated that the inclusion of the ginger-derived phyto-protease enzyme significantly enhanced body weight gain, particularly in the groups with higher enzyme levels (T3 and T4), demonstrating the enzyme's potential efficacy in improving body weight gain in birds. Except for week 1, a linear and quadratic response was observed for weekly weight gain, as well as for starter, finisher, and total weight gain in quails.

**Table 5:** Impact of ginger-derived phyto-protease on feed conversion ratio (FCR) of quail fed an APC-based diet.

Group	T1	T2	T3	T4	P value	Orthogonal contrast	
Week 1	3.24 ± 0.17	3.52 ± 0.16	3.07 ± 0.18	3.24 ± 0.14	0.1494	Linear	0.12
						Quadric	0.56
Week 2	3.40^ab^ ± 0.16	3.44^a^ ± 0.17	3.26^ab^ ± 0.15	3.21^b^ ± 0.12	0.042	Linear	0.01
						Quadric	0.01
Week 3	3.52^ab^ ± 0.17	3.63^a^ ± 0.18	3.32^bc^ ± 0.19	3.18^c^ ± 0.6	0.013	Linear	0.01
						Quadric	0.01
Starter	3.41^ab^ ± 0.20	3.53^a^ ± 0.15	3.24^bc^ ± 0.13	3.20^c^ ± 0.14	0.009	Linear	0.01
						Quadric	0.01
Week 4	3.26^ab^ ± 0.13	3.41^a^ ± 0.18	3.14^b^ ± 0.17	3.10^b^ ± 0.15	0.007	Linear	0.01
						Quadric	0.01
Week 5	3.49^ab^ ± 0.12	3.68^a^ ± 0.14	3.44^b^ ± 0.17	3.34^b^ ± 0.15	0.017	Linear	0.01
						Quadric	0.01
Week 6	3.39^ab^ ± 0.16	3.51^a^ ± 0.18	3.32^b^ ± 0.14	3.23^b^ ± 0.15	0.001	Linear	0.01
						Quadric	0.01
Finisher	3.38^b^ ± 0.14	3.51^a^ ± 0.13	3.30^bc^ ± 0.12	3.23^c^ ± 0.15	0.001	Linear	0.01
						Quadric	0.01
Total	3.39^b^ ± 0.15	3.52^a^ ± 0.12	3.28^c^ ± 0.14	3.22^c^ ± 0.18	0.001	Linear	0.01
						Quadric	0.01

Mean values bearing different superscripts in a row differ significantly ( P<0.05 ). CON: standard diet with no phyto-protease. T2: 4 % APC diet with no phyto-protease. T3: 4 % APC diet supplemented with 0.01 % ginger phyto-protease. T4: 4 % APC diet supplemented with 0.02 % ginger phyto-protease.

Table 5 presents the impact of ginger-derived phyto-protease on the feed conversion ratio (FCR) of quail fed an APC-based diet. During the first week (W1), there were no significant differences in FCR among the groups, with values ranging from 3.07 to 3.52 and a p value of 0.1494. By the second week (W2), significant differences began to emerge, with group T4 achieving the lowest FCR of 3.21, indicating more efficient feed conversion compared to group T2, which had the highest FCR of 3.44 ( p=0.0427 ).

This trend continued into the third week (W3), where T4 maintained the most efficient feed conversion (FCR of 3.18), and T2 had the least efficient feed conversion at 3.63 ( p=0.0138 ). Throughout the starter phase, significant differences were observed ( p=0.0093 ), with T4 again having the lowest FCR of 3.20 and T2 the highest at 3.53.

In the fourth week (W4), the T4 group continued to demonstrate superior feed conversion efficiency with an FCR of 3.10, while T2 exhibited the highest FCR of 3.41 ( p=0.0076 ). This pattern was evident in the fifth week (W5) and sixth week (W6) as well, with T4 showing the lowest FCRs of 3.34 and 3.23, respectively, and T2 having the highest FCRs ( p=0.0177 and p=0.0007 ).

In the finisher phase, significant differences persisted, with T4 achieving the lowest FCR of 3.23, while T2 remained the least efficient at 3.51 ( p=0.0003 ). Over the entire period, T4 consistently exhibited the best feed conversion efficiency with a total FCR of 3.22, significantly lower than T2's FCR of 3.52 ( p=0.0002 ). Except for week 1, a linear and quadratic response was observed for weekly FCR, as well as for starter, finisher, and total FCR in quails.

**Table 6:** Impact of ginger-derived phyto-protease enzyme on dressing percentage of quail fed an APC-based diet.

Group	Dressing %	Orthogonal contrast	
T1	70.00^c^ ± 1.45	Linear	0.01
		Quadratic	0.01
T2	67.00^d^ ± 1.50	Linear	0.01
		Quadratic	0.01
T3	73.00^b^ ± 1.53	Linear	0.01
		Quadratic	0.01
T4	75.00^a^ ± 1.30	Linear	0.01
		Quadratic	0.01
P value	0.0001		

Mean values bearing different superscripts in a row differ significantly ( P<0.05 ). CON: standard diet with no phyto-protease. T2: 4 % APC diet with no phyto-protease. T3: 4 % APC diet supplemented with 0.01 % ginger phyto-protease. T4: 4 % APC diet supplemented with 0.02 % ginger phyto-protease.

Table 6 shows the impact of ginger-derived phyto-protease enzyme on the dressing percentage of quail fed an APC-based diet. The dressing percentage was significantly different across the four groups. Group T4 exhibited the highest dressing percentage at 75.00 % ( ± 1.30), indicating the most efficient carcass yield among the groups. This was followed by group T3 with a dressing percentage of 73.00 % ( ± 1.53). Group T1 had a dressing percentage of 70.00 % ( ± 1.45), while group T2 had the lowest dressing percentage at 67.00 % ( ± 1.50). Orthogonal contrast analysis showed both linear and quadratic responses for dressing percentage.

**Table 7:** The impact of a phyto-protease enzyme derived from ginger on the intestinal histomorphology of quail fed APC-based diets.

Groups	Villus height ( µ m)		Villus width ( µ m)		Crypt depth ( µ m)		VH: CD	
T1	356.00^c^ ± 0.88		107.00^c^ ± 0.86		126.33^b^ ± 0.88		2.81^*c*^ ± 0.17	
T2	344.67^d^ ± 0.85		102.00^d^ ± 0.84		155.33^a^ ± 0.89		2.55^d^ ± 0.15	
T3	368.33^b^ ± 0.83		112.00^b^ ± 0.87		117.33^c^ ± 0.84		3.14^b^ ± 0.14	
T4	384.33^a^ ± 0.80		117.00^a^ ± 0.85		110.33^d^ ± 0.87		3.49^a^ ± 0.16	
P value	0.001		0.001		0.001		0.001	
Orthogonal contrast	Linear	0.01	Linear	0.01	Linear	0.01	Linear	0.01
	Quadratic	0.01	Quadratic	0.01	Quadratic	0.01	Quadratic	0.01

Mean values bearing different superscripts in a row differ significantly ( P<0.05 ). CON: standard diet with no phyto-protease. T2: 4 % APC diet with no phyto-protease. T3: 4 % APC diet supplemented with 0.01 % ginger phyto-protease. T4: 4 % APC diet supplemented with 0.02 % ginger phyto-protease.

Table 7 details the impact of a ginger-derived phyto-protease enzyme on the intestinal histomorphology of quail fed APC-based diets. The highest villus height was observed in group T4, with a mean of 384.33 µ m ( ± 0.80), significantly greater than the other groups ( p=0.000 ). Group T3 followed with 368.33 µ m ( ± 0.83), group T1 had 356.00 µ m ( ± 0.88), and the lowest height was in group T2 at 344.67 µ m ( ± 0.85). Group T4 also had the highest villus width at 117.00 µ m ( ± 0.85), significantly greater than the other groups ( p=0.000 ). This was followed by group T3 at 112.00 µ m ( ± 0.87) and group T1 at 107.00 µ m ( ± 0.86), and group T2 had the smallest width at 102.00 µ m ( ± 0.84).

Group T2 had the greatest crypt depth at 155.33 µ m ( ± 0.89), significantly deeper than the other groups ( p=0.000 ). This was followed by group T1 with 126.33 µ m ( ± 0.88) and group T3 with 117.33 µ m ( ± 0.84), and the shallowest crypts were in group T4 at 110.33 µ m ( ± 0.87). The ratio of villus height to crypt depth (VH : CD) was highest in group T4 at 3.49 ( ± 0.16), significantly better than the other groups ( p=0.000 ). Group T3 followed with a ratio of 3.14 ( ± 0.14) and group T1 with 2.81 ( ± 0.17), and the lowest ratio was in group T2 at 2.55 ( ± 0.15). A linear and quadratic response was observed in the villus dimensions of quails fed with ginger-derived protease.

**Table 8:** The effect of a phyto-protease enzyme on the nutrient digestibility of quail fed APC-based diets.

Groups	Dry matter		Crude protein		Ether extract		Crude fiber		AME Kcal kg^−1^	
T1	64.15^c^ ± 0.49		54.62^c^ ± 0.50		80.46^ab^ ± 0.65		67.78^ab^ ± 0.47		2983.3^a^ ± 0.54	
T2	62.26^d^ ± 0.48		53.85^c^ ± 0.52		79.68^b^ ± 0.61		66.49^b^ ± 0.50		2879.3^c^ ± 0.59	
T3	67.30^b^ ± 0.52		57.70^b^ ± 0.54		81.54^ab^ ± 0.67		68.58^a^ ± 0.55		2945.3^b^ ± 0.50	
T4	70.32^a^ ± 0.47		60.36^a^ ± 0.58		81.87^a^ ± 0.64		69.33^a^ ± 0.48		2957.7^b^ ± 0.53	
P value	0.001		0.001		0.019		0.032		0.001	
Orthogonal contrast	Linear	0.01	Linear	0.01	Linear	0.01	Linear	0.01	Linear	0.01
	Quadratic	0.01	Quadratic	0.01	Quadratic	0.01	Quadratic	0.01	Quadratic	0.01

Mean values bearing different superscripts in a row differ significantly ( P<0.05 ). CON: standard diet with no phyto-protease. T2: 4 % APC diet with no phyto-protease. T3: 4 % APC diet supplemented with 0.01 % ginger phyto-protease. T4: 4 % APC diet supplemented with 0.02 % ginger phyto-protease.

Table 8 illustrates the effects of a ginger-derived phyto-protease enzyme on the nutrient digestibility of quail fed APC-based diets, with a focus on dry matter, crude protein, ether extract, crude fiber, and apparent metabolizable energy (AME) in kilocalories per kilogram.

Group T4 demonstrated the highest dry matter digestibility at 70.32 % ( ± 0.47), followed by group T3 at 67.30 % ( ± 0.52) and group T1 at 64.15 % ( ± 0.49), and the lowest was in group T2 at 62.26 % ( ± 0.48). The differences between the groups were statistically significant ( p=0.0000 ), indicating a substantial improvement in dry matter digestibility with the addition of the phyto-protease enzyme.

The crude protein digestibility was significantly higher in group T4 at 60.36 % ( ± 0.58), compared to group T3 at 57.70 % ( ± 0.54) and group T1 at 54.62 % ( ± 0.50), and the lowest was in group T2 at 53.85 % ( ± 0.52). The statistical significance ( p=0.0007 ) underscores the positive effect of the enzyme on protein digestibility.

While the differences in ether extract digestibility were not statistically significant ( p=0.1591 ), group T4 had the highest percentage at 81.87 % ( ± 0.64), followed by group T3 at 81.54 % ( ± 0.67), group T1 at 80.46 % ( ± 0.65), and group T2 at 79.68 % ( ± 0.61).

Group T4 also led in crude fiber digestibility with 69.33 % ( ± 0.48), followed by group T3 at 68.58 % ( ± 0.55), group T1 at 67.78 % ( ± 0.47), and group T2 at 66.49 % ( ± 0.50). The differences among groups were statistically significant ( p=0.0326 ), suggesting that the phyto-protease enzyme aids in the breakdown of fiber.

**Table 9:** The effect of phyto-protease enzyme on the economics of broilers fed APC-based diets.

Groups	Feed cost, PKR per bird		Total cost per bird		Gross return per bird		Profit over total cost per bird	
T1	87.06^ab^ ± 0.57		127.06^ab^ ± 0.59		150.14^b^ ± 0.58		23.07^b^ ± 0.58	
T2	81.08^c^ ± 0.56		121.11^c^ ± 0.56		140.02^c^ ± 0.57		18.94^c^ ± 0.58	
T3	86.2^b^ ± 0.56		126.23^b^ ± 0.58		152.03^b^ ± 0.61		25.8^a^ ± 0.58	
T4	88.18^a^ ± 0.58		128.2^a^ ± 0.55		155.23^a^ ± 0.56		27.07^a^ ± 0.58	
P value	0.0001		0.0001		0.0000		0.0000	
Orthogonal contrast	Linear	0.01	Linear	0.01	Linear	0.01	Linear	0.01
	Quadratic	0.01	Quadratic	0.01	Quadratic	0.01	Quadratic	0.01

Total cost includes the price per bird, vaccines, feed, and supplementation price. Prices are in the currency of Pakistan (rupee).

Group T1 had the highest AME at 2983.3 Kcal kg^−1^ ( ± 0.54), although this was not significantly higher than the other groups. Group T4 had 2957.7 Kcal kg^−1^ ( ± 0.53), group T3 had 2945.3 Kcal kg^−1^ ( ± 0.50), and group T2 had the lowest AME at 2879.3 Kcal kg^−1^ ( ± 0.59). The differences were statistically significant ( p=0.0000 ), indicating that the enzyme improves energy availability from the diet. A linear and quadratic response was observed for these digestibility indices in quails fed phyto-protease enzymes.

Table 9 presents the economic analysis of feeding APC-based diets with or without ginger-derived phyto-protease enzyme. The lowest overall cost per quail was observed in the T2 group (PKR 121.08), significantly lower than the T4 group (PKR 128.18). The addition of ginger-derived phyto-protease enzyme did not significantly affect the gross return, with the highest return in the T4 group (PKR 155.23), followed by T3 (PKR 151.31), T1 (PKR 150.12), and T2 (PKR 140.02). Significant profits over total cost were noted in the T4 group (PKR 27.05), T3 group (PKR 25.09), T1 group (PKR 23.06), and T2 group (PKR 18.94). A linear and quadratic response was also observed for these parameters.

## 5 Discussion

The increase in feed intake with ginger-derived phyto-protease enzyme supplementation is closely linked to improved digestive processes. Ginger contains bioactive compounds like gingerol and shogaol, which have been shown to stimulate digestive enzyme activity, especially proteases, amylases, and lipases, improving the breakdown of feed components, particularly proteins and fats (Bakir, 2023). These enhanced enzymatic activities likely increase the palatability and digestibility of the feed, leading to higher feed consumption in supplemented groups. Additionally, phytobiotics such as ginger are known to modulate gut microbiota, which can further improve nutrient absorption and energy extraction from feed (Yang et al., 2023).

The improved body weight gain observed in ginger-supplemented groups can be attributed to several mechanisms. One key factor is the role of ginger in enhancing nutrient absorption. Studies have shown that ginger's bioactive compounds increase the efficiency of nutrient utilization by boosting the activity of intestinal enzymes and improving gut morphology (Liu et al., 2022). Increased villus height and crypt depth reduction improve the surface area for nutrient absorption, allowing for more effective uptake of amino acids, vitamins, and minerals. Moreover, ginger's antioxidant properties help mitigate oxidative stress, reducing inflammation in the gut, which may enhance the overall growth rate (Ahmed and Sharma, 2023). This reduction in oxidative stress allows for more energy to be diverted toward growth rather than maintaining cellular defense mechanisms (Chen et al., 2022).

The enhanced FCR in ginger-supplemented groups suggests more efficient nutrient utilization, which is likely due to the increased enzymatic activity facilitated by ginger-derived phyto-proteases. Gingerols and shogaols in ginger promote the secretion of digestive enzymes, improving protein and lipid digestion (Abdel-Moneim et al., 2023). As proteins are more effectively broken down into amino acids, they are better absorbed by the intestine, resulting in more efficient growth per unit of feed consumed. Additionally, the anti-inflammatory effects of ginger reduce gut inflammation, leading to improved intestinal permeability and reduced nutrient wastage (Zhang et al., 2023). The improvement in gut integrity further supports efficient nutrient absorption, as evidenced by better FCR values in the enzyme-supplemented groups (Adeyemi et al., 2022).

Ginger's influence on gut health plays a crucial role in the observed improvements in feed intake and body weight gain. Studies have shown that ginger reduces oxidative stress and inflammation in the gut by scavenging free radicals, primarily through the action of gingerol and its derivatives (Zhang et al., 2023). This antioxidative action not only preserves the structure of the intestinal lining but also enhances the efficiency of nutrient absorption. The increased villus height and reduced crypt depth observed in ginger-supplemented groups indicate enhanced nutrient absorption capacity, as these morphological changes are associated with an increase in the absorptive surface area of the gut (El-Saadony et al., 2022). Improved gut morphology is also linked to better immune function, as ginger's anti-inflammatory properties protect the gut from infections and other stressors, leading to improved overall bird health and performance (Ahmed and Sharma, 2023).

The significant improvement in dressing percentage observed in ginger-derived phyto-protease enzyme-supplemented quails, particularly in groups T3 and T4, can be attributed to enhanced nutrient digestibility, muscle development, and fat reduction. Ginger's bioactive compounds, such as gingerol and shogaol, promote the activity of digestive enzymes, improving protein digestion and absorption, which facilitates muscle growth and reduces fat deposition (Abdel-Moneim et al., 2023; Liu et al., 2022). Additionally, ginger's anti-inflammatory and antioxidant properties mitigate oxidative stress, preserving muscle integrity and promoting lean meat yield (Chen et al., 2022; Zhang et al., 2023). The enhanced gut health observed with ginger supplementation, characterized by increased villus height and reduced crypt depth, also improves nutrient absorption, leading to more efficient feed utilization and better carcass yield (Yang et al., 2023; El-Saadony et al., 2022). Consequently, the higher dressing percentage in ginger-supplemented groups highlights the enzyme's role in improving growth performance and meat quality in poultry.

In the current study, there were significant effects of a ginger-derived phyto-protease enzyme on the intestinal histomorphology of quails fed APC-based diets, showing marked variations in villus height, width, and crypt depth. The highest villus height in group T4 compared to the lowest in group T2 suggests that ginger-derived enzymes could enhance nutrient absorption by promoting villus development. This finding aligns with studies that report an increase in villus height as a response to improved intestinal health and function when phyto-protease supplements are included in diets (Ahmad et al., 2023b; Rehman et al., 2018). The significant increase in villus width in group T4 supports the hypothesis that phyto-protease enzymes promote enterocyte proliferation, contributing to enhanced digestive efficiency (Anwar et al., 2023b). Wider villi are directly linked to a larger surface area for nutrient absorption, a crucial factor in optimizing growth performance in poultry (Ahmad et al., 2023b). Crypt depth analysis showed that group T2 had the deepest crypts, which may indicate a higher turnover rate of enterocytes as a compensatory mechanism for intestinal stress or lower nutrient absorption (Mohamed and Hassan, 2023). This response is consistent with findings that deeper crypts often correlate with increased cellular activity required for gut repair and renewal under suboptimal dietary conditions (Hassan et al., 2021). The VH : CD ratio, a key indicator of gut health, was highest in group T4, indicating a better absorptive capacity and reduced intestinal cell turnover (Akhtar et al., 2023). A higher VH : CD ratio generally reflects a well-maintained intestinal structure that favors nutrient uptake and overall gut functionality (Ali et al., 2019). The observed linear and quadratic responses in villus dimensions further emphasize the dose-dependent impact of ginger-derived proteases on gut morphology, supporting previous findings on the beneficial role of phytogenic additives in poultry nutrition (Khan et al., 2024).

## 6 Conclusions

These results indicate that the optimal inclusion level of ginger-derived phyto-protease enzyme for enhancing growth performance, feed efficiency, carcass quality, and gut health in Japanese quails is the highest concentration tested in this study (0.02 %).

## Data Availability

No data sets were used in this article.
